# Supplementary material for: OpenMM 7: Rapid development of high performance algorithms for molecular dynamics
Source: PLoS Comput Biol. 2017 Jul 26;13(7):e1005659. doi: 10.1371/journal.pcbi.1005659 (PMC5549999; doi:10.1371/journal.pcbi.1005659)
Supplement: S1 Source Code — (ZIP) [file pcbi.1005659.s002.zip › OpenMM-7.0.1-Source/docs/api-c++/generated/OpenMM.SerializationNode.html]

SerializationNode — OpenMM @OPENMM\_MAJOR\_VERSION@.@OPENMM\_MINOR\_VERSION@ documentation


# SerializationNode¶

*class* `OpenMM::``SerializationNode`¶
:   A `SerializationNode` stores information about an object during serialization or deserialization.

    When an object is serialized, its `SerializationProxy` is first called to copy information about the object into a `SerializationNode`. That information can then be written to the output stream in the desired format.

    When an object is deserialized, the input stream is read and the information is stored into a `SerializationNode`. The appropriate `SerializationProxy` is then called to reconstruct the object.

    SerializationNodes are arranged in a tree. There will often be a one-to-one correspondence between objects and SerializationNodes, but that need not always be true. A proxy is free to create whatever child nodes it wants and store information in them using whatever organization is most convenient.

    Each `SerializationNode` can store an arbitrary set of “properties”, represented as key-value pairs. The key is always a string, while the value may be a string, an int, or a double. If a value is specified using one data type and then accessed as a different data type, the node will attempt to convert the value in an appropriate way. For example, it is always reasonable to call `getStringProperty()` to access a property as a string. Similarly, you can use `setStringProperty()` to specify a property and then access it using `getIntProperty()`. This will produce the expected result if the original value was, in fact, the string representation of an int, but if the original string was non-numeric, the result is undefined.

    Methods

    |  |  |
    | --- | --- |
    | `getName` | Get the name of this `SerializationNode`. |
    | `setName` | Set the name of this `SerializationNode`. |
    | `getChildren` | Get a reference to this node’s child nodes. |
    | `getChildren` | Get a reference to this node’s child nodes. |
    | `getChildNode` | Get a reference to the child node with a particular name. |
    | `getChildNode` | Get a reference to the child node with a particular name. |
    | `getProperties` | Get a map containing all of this node’s properties. |
    | `hasProperty` | Determine whether this node has a property with a particular node. |
    | `getStringProperty` | Get the property with a particular name, specified as a string. |
    | `getStringProperty` | Get the property with a particular name, specified as a string. |
    | `setStringProperty` | Set the value of a property, specified as a string. |
    | `getIntProperty` | Get the property with a particular name, specified as an int. |
    | `getIntProperty` | Get the property with a particular name, specified as an int. |
    | `setIntProperty` | Set the value of a property, specified as an int. |
    | `getBoolProperty` | Get the property with a particular name, specified as an bool. |
    | `getBoolProperty` | Get the property with a particular name, specified as a bool. |
    | `setBoolProperty` | Set the value of a property, specified as a bool. |
    | `getDoubleProperty` | Get the property with a particular name, specified as a double. |
    | `getDoubleProperty` | Get the property with a particular name, specified as a double. |
    | `setDoubleProperty` | Set the value of a property, specified as a double. |
    | `createChildNode` | Create a new child node |
    | `createChildNode` | Create a new child node by serializing an object. |
    | `decodeObject` | Reconstruct an object based on the information stored in this node. |

    *const* std::string &`getName`() *const*¶
    :   Get the name of this `SerializationNode`.

    void `setName`(*const* std::string &*name*)¶
    :   Set the name of this `SerializationNode`.

        |  |  |
        | --- | --- |
        | Parameters: |  |

        - **name** – the new name of the `SerializationNode`

    *const* std::vector<SerializationNode> &`getChildren`() *const*¶
    :   Get a reference to this node’s child nodes.

    std::vector<SerializationNode> &`getChildren`()¶
    :   Get a reference to this node’s child nodes.

    *const* SerializationNode &`getChildNode`(*const* std::string &*name*) *const*¶
    :   Get a reference to the child node with a particular name. If there is no child with the specified name, this throws an exception.

        |  |  |
        | --- | --- |
        | Parameters: |  |

        - **the** – name of the child node to get

    SerializationNode &`getChildNode`(*const* std::string &*name*)¶
    :   Get a reference to the child node with a particular name. If there is no child with the specified name, this throws an exception.

        |  |  |
        | --- | --- |
        | Parameters: |  |

        - **the** – name of the child node to get

    *const* std::map<std::string, std::string> &`getProperties`() *const*¶
    :   Get a map containing all of this node’s properties.

    bool `hasProperty`(*const* std::string &*name*) *const*¶
    :   Determine whether this node has a property with a particular node.

        |  |  |
        | --- | --- |
        | Parameters: |  |

        - **name** – the name of the property to check for

    *const* std::string &`getStringProperty`(*const* std::string &*name*) *const*¶
    :   Get the property with a particular name, specified as a string. If there is no property with the specified name, an exception is thrown.

        |  |  |
        | --- | --- |
        | Parameters: |  |

        - **name** – the name of the property to get

    *const* std::string &`getStringProperty`(*const* std::string &*name*, *const* std::string &*defaultValue*) *const*¶
    :   Get the property with a particular name, specified as a string. If there is no property with the specified name, a default value is returned instead.

        |  |  |
        | --- | --- |
        | Parameters: |  |

        - **name** – the name of the property to get
        - **defaultValue** – the value to return if the specified property does not exist

    SerializationNode &`setStringProperty`(*const* std::string &*name*, *const* std::string &*value*)¶
    :   Set the value of a property, specified as a string.

        |  |  |
        | --- | --- |
        | Parameters: |  |

        - **name** – the name of the property to set
        - **value** – the value to set for the property

    int `getIntProperty`(*const* std::string &*name*) *const*¶
    :   Get the property with a particular name, specified as an int. If there is no property with the specified name, an exception is thrown.

        |  |  |
        | --- | --- |
        | Parameters: |  |

        - **name** – the name of the property to get

    int `getIntProperty`(*const* std::string &*name*, int *defaultValue*) *const*¶
    :   Get the property with a particular name, specified as an int. If there is no property with the specified name, a default value is returned instead.

        |  |  |
        | --- | --- |
        | Parameters: |  |

        - **name** – the name of the property to get
        - **defaultValue** – the value to return if the specified property does not exist

    SerializationNode &`setIntProperty`(*const* std::string &*name*, int *value*)¶
    :   Set the value of a property, specified as an int.

        |  |  |
        | --- | --- |
        | Parameters: |  |

        - **name** – the name of the property to set
        - **value** – the value to set for the property

    bool `getBoolProperty`(*const* std::string &*name*) *const*¶
    :   Get the property with a particular name, specified as an bool. If there is no property with the specified name, an exception is thrown.

        |  |  |
        | --- | --- |
        | Parameters: |  |

        - **name** – the name of the property to get

    bool `getBoolProperty`(*const* std::string &*name*, bool *defaultValue*) *const*¶
    :   Get the property with a particular name, specified as a bool. If there is no property with the specified name, a default value is returned instead.

        |  |  |
        | --- | --- |
        | Parameters: |  |

        - **name** – the name of the property to get
        - **defaultValue** – the value to return if the specified property does not exist

    SerializationNode &`setBoolProperty`(*const* std::string &*name*, bool *value*)¶
    :   Set the value of a property, specified as a bool.

        |  |  |
        | --- | --- |
        | Parameters: |  |

        - **name** – the name of the property to set
        - **value** – the value to set for the property

    double `getDoubleProperty`(*const* std::string &*name*) *const*¶
    :   Get the property with a particular name, specified as a double. If there is no property with the specified name, an exception is thrown.

        |  |  |
        | --- | --- |
        | Parameters: |  |

        - **name** – the name of the property to get

    double `getDoubleProperty`(*const* std::string &*name*, double *defaultValue*) *const*¶
    :   Get the property with a particular name, specified as a double. If there is no property with the specified name, a default value is returned instead.

        |  |  |
        | --- | --- |
        | Parameters: |  |

        - **name** – the name of the property to get
        - **defaultValue** – the value to return if the specified property does not exist

    SerializationNode &`setDoubleProperty`(*const* std::string &*name*, double *value*)¶
    :   Set the value of a property, specified as a double.

        |  |  |
        | --- | --- |
        | Parameters: |  |

        - **name** – the name of the property to set
        - **value** – the value to set for the property

    SerializationNode &`createChildNode`(*const* std::string &*name*)¶
    :   Create a new child node

        |  |  |
        | --- | --- |
        | Parameters: |  |

        - **name** – the name of the new node to create

        |  |  |
        | --- | --- |
        | Returns: | a reference to the newly created node |

    `template <class T>`

    SerializationNode &`createChildNode`(*const* std::string &*name*, *const* T \**object*)¶
    :   Create a new child node by serializing an object. A `SerializationProxy` is automatically selected based on the object’s type, then invoked to populate the newly created node.

        Note that, while this method is templatized based on the type of object being serialized, the typeid() operator is used to select the proxy. This means the template argument may be a base class, and the correct proxies will still be selected for objects of different subclasses.

        |  |  |
        | --- | --- |
        | Parameters: |  |

        - **name** – the name of the new node to create
        - **object** – a pointer to the object to serialize

        |  |  |
        | --- | --- |
        | Returns: | a reference to the newly created node |

    `template <class T>`

    T \*`decodeObject`() *const*¶
    :   Reconstruct an object based on the information stored in this node. A `SerializationProxy` is automatically selected based on the information stored in the node, then it is invoked to create the object.

        The template parameter may be either the actual type of the object, or any base class to which it may be cast.

        |  |  |
        | --- | --- |
        | Returns: | a pointer to the newly created object. The caller assumes ownership of the object. |

High performance molecular simulation on GPUs


### Navigation

- Core Objects
- Forces
- Integrators
- Extras

©2015, Stanford University and the Authors.
|
Powered by Sphinx 1.3.1
& Alabaster 0.7.7
|
Page source
